# AS-OCTA–Guided Versus Slit Lamp-Guided Laser Peripheral Iridotomy for Primary Angle-Closure Suspect Patients: A Short-Term Result

**DOI:** 10.1167/tvst.14.11.2

**Published:** 2025-11-03

**Authors:** Jingjing Lin, Jianbo Mao, Meihui He, Yun Zhang, Li Nie

**Affiliations:** 1 National Clinical Research Center of Ocular Diseases, Eye Hospital, Wenzhou Medical University, Wenzhou, People's Republic of China; 2 Department of Optometry and Vision Sciences, The University of Melbourne, Melbourne, Victoria, Australia; 3 Zhejiang Provincial People’s Hospital, Hangzhou City, Zhejiang Province, People's Republic of China

**Keywords:** anterior segment OCT angiography, glaucoma, laser peripheral iridotomy

## Abstract

**Purpose:**

This study aimed to explore the possibility of the clinical application using anterior segment optical coherence tomography angiography (AS-OCTA)-guided laser peripheral iridotomy (LPI).

**Methods:**

AS-OCT/OCTA was performed before LPI and at 1 hour, 1 day, and 1 week post-LPI. All the right eyes of patients with primary angle-closure suspect (PACS) were assigned to the AS-OCTA–guided group, in which the peripheral site with the sparsest iris vasculature on AS-OCTA images was selected for LPI. The left eyes underwent LPI in the slit lamp-guided group, where the site was chosen based on the presence of an iris crypt by clinicians using the slit lamp. The two groups were compared for the incidence of anterior chamber bleeding observed by the LPI operator, anterior chamber particle (ACP) index, mean angle opening distance (AOD750), and anterior chamber depth (ACD) measured from AS-OCT images, as well as the vessel density and perfusion area obtained from AS-OCTA images.

**Results:**

A total of 30 patients with PACS were included in this study. The incidence of anterior chamber bleeding during LPI was 13.33% in the AS-OCTA–guided group, compared with 43.33% in the slit lamp-guided group (*P* = 0.010). The prominent difference was observed 1 hour after LPI, with the AS-OCTA-guided group showing significantly lower vessel density (*P* = 0.028), perfusion area (*P* = 0.003), and ACP (*P* = 0.004), but a larger AOD750 (*P* < 0.001) compared with the slit lamp-guided group.

**Conclusions:**

Utilizing AS-OCTA to guide the LPI procedure can significantly decrease the incidence of anterior chamber bleeding and mitigate inflammation.

**Translational Relevance:**

This study shows that the AS-OCTA–guided LPI procedure could bridge advanced imaging technology and clinical glaucoma management.

## Introduction

Primary angle closure glaucoma (PACG) is a leading blinding disease worldwide characterized by glaucomatous changes in the optic disc and visual field secondary to acute or chronic elevated intraocular pressure (IOP).^1^ In natural history, people with primary angle closure suspect (PACS) demonstrate a trajectory toward primary angle closure (PAC) and PACG.^1^ Since 1994, laser peripheral iridotomy (LPI) has served as an effective method for the management of PACG,^2^^–^^4^ as it facilitates the opening of the anterior chamber angle, eliminates pupillary block, and eventually reduces the chance of angle closure. Indeed, LPI has a prophylactic effect in reducing progression from PACS to PACG. However, LPI has several complications, including IOP spike,^5^ anterior chamber bleeding,^6^^,^^7^ and dysphotopsia.^8^ Anterior chamber bleeding during LPI has been reported to be up to 41%,^6^ leading to an inflammatory reaction in the anterior chamber, elevated IOP, and blurred vision. Destroyed vascular structures by LPI in the iris could be one of the reasons. Normally, clinicians use slit lamp biomicroscopy to identify the laser location (usually in iris crypt,^9^^,^^10^ a site considered to have a thinner stoma and sparse vessels) and adjust the laser settings when performing LPI. As such, the subjective judgment of laser placement purely by clinicians remains a concern, particularly when no obvious iris crypt is visible under slit lamp examination.

On the other hand, approaches such as ultrasound biomicroscopy (UBM), gonioscopy, and anterior segment optical coherence tomography (AS-OCT) are often performed prior to and after the LPI procedure to evaluate anatomic changes in the anterior segmentation, whereas few report these imaging approaches in guiding the site of LPI treatment. Recently, OCT angiography (OCTA) has been used to visualize the iris vasculature noninvasively.^10^ Moreover, swept-source OCT (SS-OCT) devices with a longer wavelength enable deeper penetration and a more comprehensive view of the iris vessels and the anterior segment.^11^^–^^13^ Until now, whether AS-OCTA guided LPI improves clinicians’ ability to identify an optimal and non-vascular site for LPI, thereby reducing the risk of bleeding, or whether it provides a comparable effect to slit lamp-assisted LPI in opening the anterior chamber remains understudied.

Collectively, this study aimed to assess the clinical utility of AS-OCTA in guiding the LPI procedure by comparing the incidence of anterior chamber bleeding, vascular alterations in the iris, anterior inflammatory reactions, and anatomic changes between AS-OCTA–guided LPI and slit lamp-guided LPI in eyes with PACS.

## Methods

### Ethics Statement

This study was approved by the Institutional Review Board and Ethics Committee of the Eye Hospital of Wenzhou Medical University (No. H2023-036-K-34) and adhered to the tenets of the Declaration of Helsinki. Written informed consent for the LPI procedure was obtained from the patients after a detailed explanation of the nature, benefits, and possible consequences.

### Subjects and Examination Methods

All patients were recruited at the glaucoma outpatient department of the Eye Hospital of Wenzhou Medical University from June 2023 to July 2023. A complete eye examination was performed by a glaucoma specialist (author L.N.) via slit lamp (Topcon SL-1E; Topcon, Tokyo, Japan) first, and then followed by ocular tests, including best corrected visual acuity (BCVA), IOP by noncontact tonometer (TOPCON CT-800; Topcon, Tokyo, Japan), optical nerve and macular assessments by ocular fundus photography (Canon CX-1; Canon Inc, Tokyo, Japan), and OCT (Spectralis OCT; Heidelberg Engineering, Heidelberg, Germany), and anatomic assessments in the anterior segment by UBM and gonioscopy.

Patients with PACS in both eyes were included in this study. According to the International Society of Geographic and Epidemiologic Ophthalmology (ISGEO) protocol,^14^ PACS was defined as an eye exhibiting ≥180 degrees appositional contact between the peripheral iris and the pigmented posterior trabecular meshwork in the primary position without indentation during gonioscopy, within normal IOP (<21 millimeters of mercury [mm Hg]), and with the absence of peripheral anterior synechiae and glaucomatous optic neuropathy. The exclusion criteria were as follows: (1) BCVA (expressed as decimal) in either eye <0.8; (2) history of ocular surgery; (3) history of ocular disease that may affect the iris or BCVA, such as uveitis, iris tumor, diabetic retinopathy, severe cataract, or macular degeneration; (4) previous treatments for glaucoma, including drugs, laser, or surgery; and (5) AS-OCT/OCTA image quality <7.

**Figure 1.**
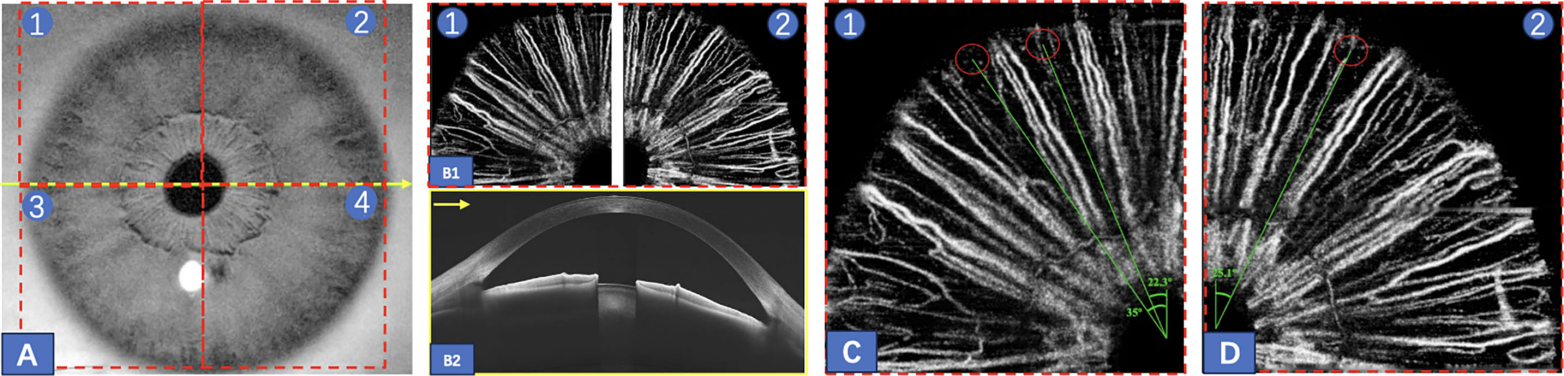
SS-OCT/OCTA images of the iris and anterior chamber. (**A**) An infrared image of the iris with 6 mm * 6 mm vascular scans performed in each of the 4 iris quadrants; (**B1**) vessel structures in the superior iris quadrants; (**B2**) tomography of the anterior chamber; (**C**) and (**D**). Non-vascularized locations (*red circles*) and their corresponding angles relative to the right horizontal meridian (125 degrees, 112.3 degrees, and 63.9 degrees), identifying candidate locations for the LPI.

After one drop of pilocarpine nitrate 0.5% eye drops (Shandong Bausch & Lomb Freda Pharmaceutical Co., Ltd., Jinan, China) was administered 30 minutes prior to the treatment to induce miosis and thin the iris, AS-OCT/OCTA assessment was performed by a trained glaucoma doctor (author J.L.). Horizontal anterior chamber scans were acquired using AS-OCT (Fig. 1B2), which possessed a swept-source laser with a central wavelength of 1050 nm and a scanning speed of 200 kilohertz (kHz; VG200S; SVision Imaging, Henan, China).^15^^,^^16^ The axial resolution, lateral resolution, and scan depth (self-set) were 5, 13 µm, and 8.5 mm, respectively. Each B-scan with a length of 18 mm and was overlapped 64 times. Iris vascular images were obtained using AS-OCTA (Fig. 1B1). In AS-OCTA images, a 6 mm * 6 mm vascular scan was performed in each of the 4 iris quadrants (Fig. 1A).

Based on the choice of laser spot, eyes were divided into two groups: the AS-OCTA-guided group (right eyes) and the slit lamp-guided group (left eyes). For the AS-OCTA–guided group, the operator (author J.L.) marked all non-vascularized areas in the peripheral iris and recorded each location as an absolute value between 0 degrees and 180 degrees, measured relative to the superior vertical meridian (Figs. 1C, 1D). In these eyes, AS-OCTA was used to identify the iris crypt with the sparsest vasculature. In the slit lamp-guided group, laser spot selection was determined by the operator using slit lamp biomicroscopy. The preferred spot was within an iris crypt from 11 o'clock to 1 o'clock; other sites would be considered if no crypts existed in the superior region.

All LPI were performed by one operator (author N.L.) using an Abraham lens (Ocular Abraham Iridectomy YAG Laser Lens; Ocular Instruments Inc., Bellevue, WA) under a neodymium: yttrium–aluminum–garnet (Nd: YAG) laser (Visulas YAG III; Carl Zeiss Meditec, Dublin, CA), with an initial laser setting of 3.5 mJ, a single pulse per burst, and a spot size of 50 µm. Digital pressure was applied to the contact lens if bleeding occurred during the procedure to achieve hemostasis.

Anterior iris images were captured immediately after LPI treatment through the camera equipped on the slit lamp. The number of laser pulses, average energy, actual laser location (expressed with an absolute angle), and the presence of any anterior bleeding were observed and recorded. Following the LPI, fluorometholone 0.1% eye drops (Santen Pharmaceutical Co., Ltd., Shiga Plant, Osaka, Japan) were instilled 3 times daily for 1 week. Patients with normal IOP 1 hour after LPI were discharged at the outpatient department, whereas brimonidine 0.15% eye drops (Novartis Europharm Limited, London, UK) would be prescribed if IOP >25 mm Hg, and Timolol Maleate 0.5% eye drops (Wujing Pharmaceutical Co., Ltd., Wuhan, China) was added if IOP >30 mm Hg.

All patients were re-assessed at the outpatient department and underwent IOP measurement and AS-OCT/OCTA imaging at 1 day and 1 week after LPI. All AS-OCT/OCTA examinations performed after the LPI procedure were conducted by the same experienced operator (author J.L.) under myotic conditions induced by either 0.5% pilocarpine nitrate eye drops or strong light exposure to the pupil.

### Quantification in AS-OCT/OCTA Images

**Figure 2.**
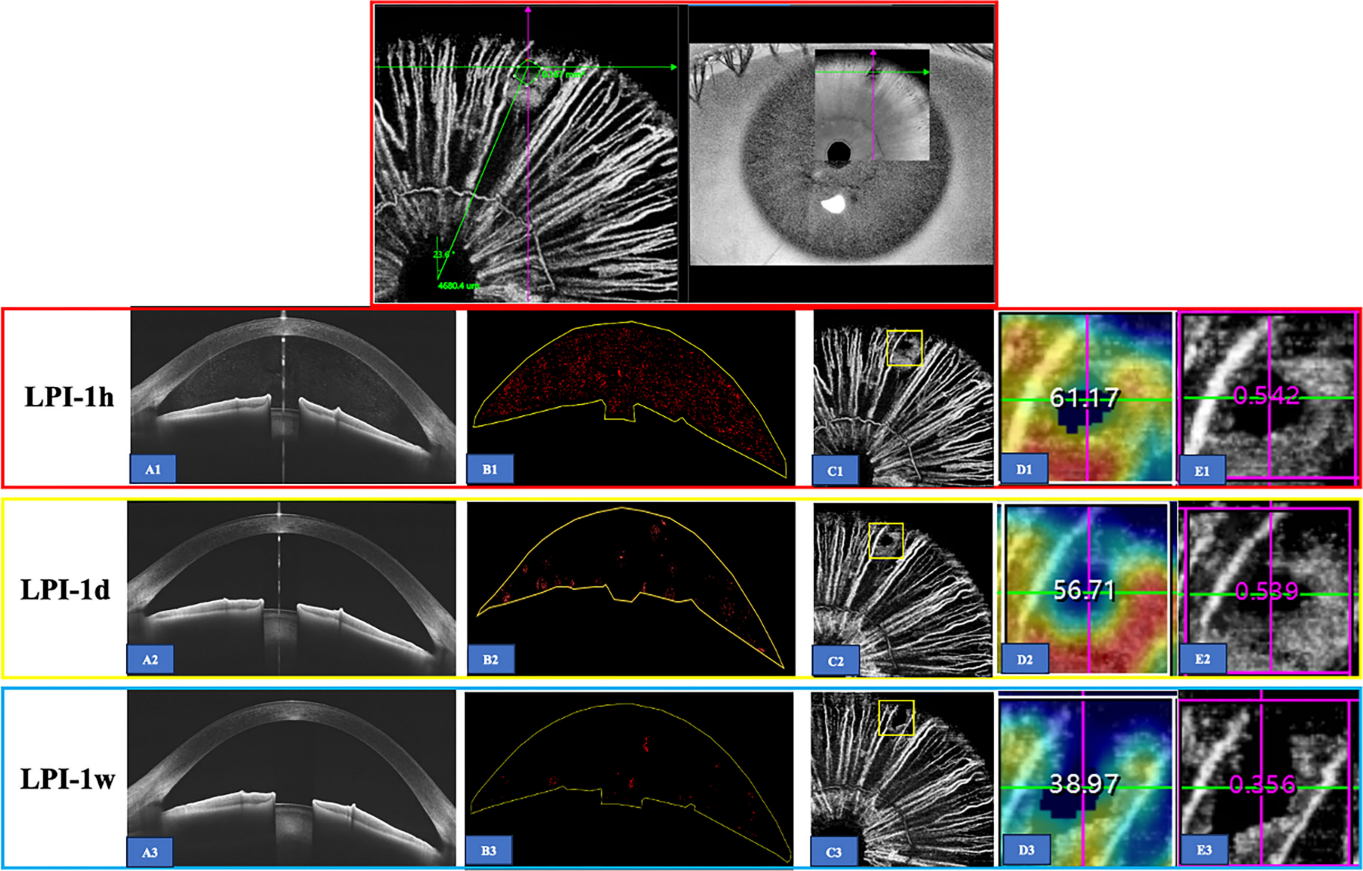
AS-OCT image of a 54-year-old female patient, whose right eye had undergone LPI 1 week earlier. (**A1–A3**) demonstrated the anterior chamber in AS-OCT images that obtained from horizontal single line scans. Findings at LPI 1 hour (*red rectangle*), LPI 1 day (*yellow rectangle*), and LPI 1 week (*blue rectangle*) post-LPI were shown. The LPI location was at 66.4 degrees from the right horizontal meridian, with a size area of 0.167 mm^2^; (**B1–B3**) showed output of the “Bernsen” algorithm in the “auto local threshold” to identify particles in the anterior chamber. (**D1–D3**) and (**E1–E3**) represented vessel density and perfusion area in a 1-mm diameter region centered on the LPI site (*yellow square* in **C1–C3**), calculated using the built-in software (version 3.0.115).

Using the built-in software (version 3.0.115), the anterior chamber depth (ACD) and the angle opening distance (AOD750) were obtained from horizontal AS-OCT images. The AOD750 was defined as the perpendicular distance from the trabecular meshwork at 750 µm anterior to the scleral spur to the anterior iris surface. To objectively quantify aqueous flare and cells in the anterior chamber after LPI, AS-OCT images were binarized first and then thresholded using the “Bernsen” algorithm in the “auto local threshold” mode with ImageJ software (version 1.53K; National Institutes of Health, Bethesda, MD; available at: http://imagej.nih.gov/ij/; Figs. 2 B1–B3).^17^ The densities of the particles in the anterior chamber were quantified and expressed as an anterior chamber particle (ACP) index. Two trained clinicians (authors J.L. and L.N.) processed the images and completed the ACP quantification, and the final value was calculated as the average.

In AS-OCTA images, the vessel density and the perfusion area in a 1-mm diameter region centered on the LPI site were calculated (Figs. 2C1–C3, 2D1–D3, 2E1–E3) through built-in algorithms. Vessel density was defined as the percentage of the region of interest (ROI) occupied by blood vessels, whereas the perfusion area was defined as the total area within the ROI showing detectable blood flow signals. In our study, the ROI was set to the system's smallest measurement scale, which was 1 mm × 1 mm. The exact LPI site’s angle and size were evaluated in the 1-hour image after LPI (Fig. 2, top layer).

### Statistical Analysis

All statistical comparisons between the AS-OCTA-guided and slit lamp-guided groups were conducted using SPSS software (version 26.0; SPSS, Chicago, IL) and GraphPad Prism (version 9.5.1; GraphPad Software, San Diego, CA). The Shapiro-Wilk test was used to assess normality. All parametric data were expressed as mean ± standard deviation (SD), whereas nonparametric data were shown as median and interquartile ranges (IQRs; 25th percentiles to 75th percentiles). Between the two groups, we applied a paired *t*-test for parameters with normal distributions; otherwise, the Mann-Whitney *U* test was used. Categorical data was analyzed with a chi-square test. Because both eyes of the same patient were compared, we performed generalized estimating equations (GEEs) among repeated variables. Inter-assessor agreement (between authors J.L. and L.N.) on the measurement of ACP was calculated with an intraclass correlation coefficient (ICC). Correlations between continuous variables were analyzed with Pearson's correlation analysis; otherwise, Spearman's correlation was used. A *P* value < 0.05 was statistically significant.

## Results

Thirty patients with PACS in both eyes were included in this study. There were 4 male patients and 26 female patients, and the average age was 57.67 ± 10.40 years. As presented in Table 1, there was no significant difference between the two groups in the laser parameters (total dots, average energy, location [superior], and LPI size), nor IOP results assessed before or after LPI (all *P* > 0.05). Surprisingly, the incidence of anterior chamber bleeding was significantly lower in the AS-OCTA–guided group (13.33% vs. 43.33%, *P* = 0.010). At 1 hour after LPI, 7 eyes in the AS-OCTA–guided group had an IOP spike (an elevation of 8 mm Hg increase from baseline), 6 of which received brimonidine 0.15%. In the slit lamp-guided group, nine eyes suffered an IOP spike and then the patient received drug treatment. There was no difference in the number of drugs prescribed after LPI between the two groups (*P* = 0.371). None of the patients complained of blurred vision or any visual symptoms 1 day or 1 week after the LPI.

**Table 1.** Basic Characteristics and Laser Parameters of Patients

Variables	OD	OS	Statistical Value	*P* Value
Patients, *n*	30			
Sex, male/female, *n*	4/26		−	
Age, y, mean ± SD	57.67 ± 10.40			
IOP,^a^ mm Hg, mean ± SD				
Pre-LPI	15.06 ± 3.73	14.79 ± 3.00	−0.763	0.452
Post-LPI in 1 h	18.76 ± 7.31	19.30 ± 8.01	−0.593	0.558
Post-LPI in 1 d	12.25 ± 2.91	13.70 ± 5.03	−1.412	0.171
Post-LPI in 1 wk	13.84 ± 3.57	13.50 ± 2.66	0.569	0.574
Laser parameters				
Total dots^b^ (n, median (IQR) )	24 (10.75–45.50)	25 (15.25–37.50)	0.465	0.642
Average energy,^b^ mJ, median (IQR)	3.8 (3.6–4.0)	3.8 (3.6–4.0)	−1.000	0.317
Location,^c^ superior, *n*	24	18	2.857	0.091
Size,^b^ mm^2^, median (IQR)	0.26 (0.21–0.33)	0.27 (0.23–0.31)	−0.43	0.965
Anterior chamber bleeding,^c^ %	13.33% (4/30)	43.33% (13/30)	6.648	0.010

a Paired *t*-test.

b Mann-Whitney *U* test.

c Chi-square test.

**Figure 3.**
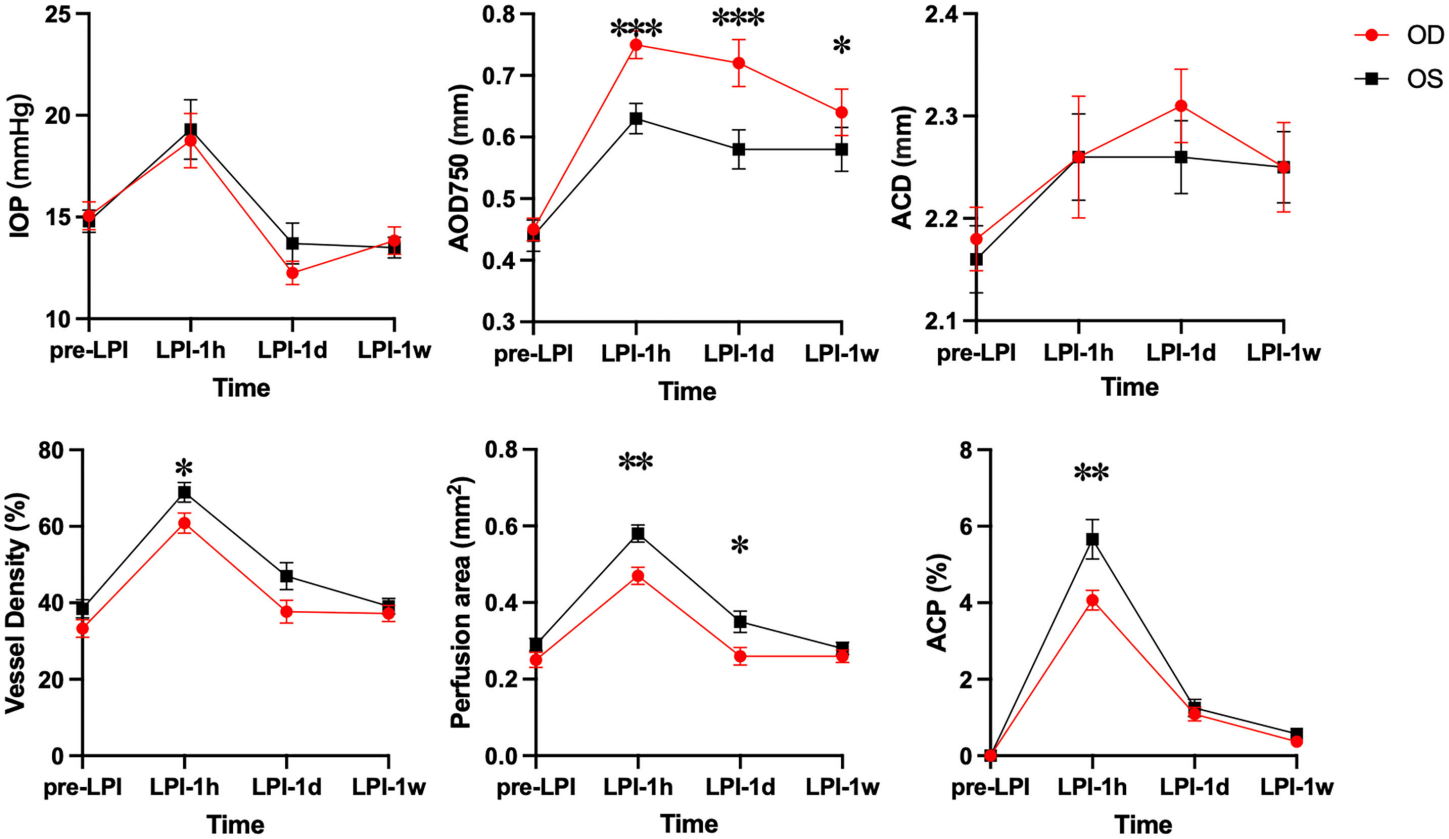
IOP, AS-OCT, and AS-OCTA parameters between the AS-OCTA-guided group and the slit lamp-guided group. LPI 1 hour, LPI 1 day, and LPI 1 week represent post-LPI in 1 hour, 1 day, and 1 week, respectively. *P < 0.05; **P < 0.01; ***P < 0.001. Vessel density and perfusion area were quantified in a 1-mm diameter region centered on the LPI site. ACP, anterior chamber particle index; AOD750, angle opening distance 750 µm from the scleral spur; ACD, anterior chamber depth. All parameters were presented as mean ± SEM.

As shown in Table 2, the vessel density and perfusion area altered significantly over time in both groups (*P* < 0.001). The slit lamp-guided group had a significantly larger perfusion area than the AS-OCTA-guided group (*P* = 0.012), whereas vessel density did not differ significantly between the two groups (*P* = 0.081). Post-analysis revealed no significant differences in vessel density or perfusion area between the groups prior to LPI or 1 week after LPI (all *P* > 0.05). Compared with the slit lamp-guided group, the AS-OCTA-guided group had significantly lower vessel density at 1 hour after LPI and a significantly smaller perfusion area at both 1 hour and 1 day after LPI (all *P* < 0.05; Fig. 3).

**Table 2.** *P* Values Among AS-OCT/AS-OCTA Parameters Between Two Groups Before LPI and After LPI in 1 Hour, 1 Day, and 1 Week

	Vessel Density, %	Perfusion Area, mm^2^	ACP, %	AOD750, mm	ACD, mm
OD			−		
Pre-LPI	33.27 ± 12.92	0.25 ± 0.11		0.45 ± 0.10	2.18 ± 0.17
Post-LPI in 1 h	60.88 ± 14.34	0.47 ± 0.12	4.07 ± 1.38	0.75 ± 0.12	2.26 ± 0.31
Post-LPI in 1 d	37.73 ± 14.28	0.26 ± 0.11	1.09 ± 0.81	0.72 ± 0.18	2.31 ± 0.16
Post-LPI in 1 wk	37.21 ± 11.43	0.26 ± 0.09	0.37 ± 0.35	0.64 ± 0.17	2.25 ± 0.24
OS			−		
Pre-LPI	38.42 ± 13.18	0.29 ± 0.09		0.44 ± 0.14	2.16 ± 0.18
Post-LPI in 1 h	68.91 ± 14.06	0.58 ± 0.12	5.66 ± 2.74	0.63 ± 0.13	2.26 ± 0.22
Post-LPI in 1 d	46.95 ± 16.76	0.35 ± 0.13	1.25 ± 1.02	0.58 ± 0.15	2.26 ± 0.16
Post-LPI in 1 wk	39.13 ± 11.34	0.28 ± 0.09	0.57 ± 0.57	0.58 ± 0.16	2.25 ± 0.19
*P* value					
*P*1	0.000	0.000	0.000	0.000	0.000
*P*2	0.081	0.012	0.000	0.033	0.767
*P*3	0.089	0.028	0.000	0.008	0.747

ACD, anterior chamber depth; ACP, anterior chamber particle, expressed as an index of the area of the signals from the particles divided by the area of the anterior chamber; AOD750, angle opening distance 750 µm from the scleral spur.

*P*1, *P*2, and *P*3 represent the *P* values among time, group, and time * group, respectively.

Vessel density and perfusion area were located in a 1-mm diameter region centered on the LPI site, acquired by built-in software in AS-OCTA (VG200S; SVision Imaging, Henan, China).

All values were presented as mean ± standard deviation.

A significantly higher ACP index was found in the slit lamp-guided group than the AS-OCTA-guided group at 1 hour after LPI (5.66 ± 2.74% vs. 4.07 ± 1.38%, *P* = 0.004). Moreover, there was excellent inter-assessor agreement between the two assessors regarding ACP values, as evidenced by a high ICC (0.966, 95% confidence interval [CI] = 0.954–0.975).

In Table 2, both AOD750 and ACD changed significantly over time in both groups (*P* < 0.001). The AS-OCTA-guided group showed significantly higher AOD750 values (*P* = 0.03), but similar ACD values (*P* > 0.05) compared with the slit lamp-guided group. As shown in Figure 3, the AS-OCTA-guided group had a larger AOD750 than the slit lamp-guided group at 1 hour, 1 day, and 1 week (all *P* < 0.05). No significant differences in ACD were observed at any time point (*P* > 0.05).

### Correlation Analysis

No significant correlations were found among laser parameters, IOPs, or the incidence of bleeding. However, higher numbers of laser spots resulted in a higher ACP index 1 hour after the LPI (*r* = 0.262, *P* = 0.043). The vessel density and perfusion area were significantly correlated with average LPI energy (*r* = 0.296, *P* = 0.022 and *r* = 0.272, *P* = 0.036) at 1 week. There was also a negative correlation between the perfusion area and LPI size (*r* = −0.334, *P* = 0.009), as well as between the vessel density and the incidence of anterior chamber bleeding (*r* = −0.287, *P* = 0.026).

## Discussion

In this study, we investigated the potential of using AS-OCTA to assist with clinical LPI procedures. There were some key results: first, the incidence of anterior chamber bleeding during LPI guided by AS-OCTA was significantly lower compared with slit lamp-guided LPI (13.33% vs. 43.33%). Second, AS-OCTA-guided LPIs reduced anterior chamber inflammatory responses and local vascular changes around the LPI site. Third, AS-OCT-guided LPI returned a significantly larger angle opening in the peripheral chamber.

In our study, the AS-OCTA images provided a clear view of the iris vasculature (see Figs. 1B2, 1C, 1D), thus guiding to identification of those LPI sites that were least vascularized to minimize the risk of bleeding. On the other hand, the current practice is to use slit lamp biomicroscopy to select iris crypts in the superior region; however, this approach does not provide clinicians with information on the extent of vascularization within the crypts. Particularly when no obvious crypts are present in the superior region or any region, it becomes difficult for clinicians to identify an appropriate LIP site. The incidence of bleeding in slit lamp-guided LPIs was similar to previous reports, which ranged from 30% to 41%.^6^ Of note, some researchers^7^ assessed and graded the anterior chamber bleeding to 4 levels: 0 indicated no bleeding; +1, minor bleeding stopped by pressure of the lens; +2, bleeding not controlled by force, without macroscopic hyphema; and +3, macroscopic hyphema. However, all eyes in our study were confined to grades 0 and +1, so such a system was not applicable to semi-quantify the bleeding extent in this study.

After the LPI procedure, the acute and transient IOP elevation is the most common complication, as the ratio of IOP spike has been reported up to 10%.^6^^,^^18^ In our study, the slit lamp-guided group had more IOP spikes than the AS-OCTA–guided group 1 hour after LPI (9 eyes vs. 7 eyes, *P* = 0.77). This result could be secondary to bleeding, as it induced elevated IOP by releasing blood cells and debris that impeded aqueous drainage. In addition, this study also showed that ACP was significantly lower with AS-OCTA guidance compared with the non-guided approach at 1 hour after LPI (*P* < 0.01), indicating fewer inflammatory cells, blood cells, and iris pigmentary debris. This difference in ACP was absent at 1 day and 1 week, which might be due to the administration of fluorometholone 0.1% which suppressed inflammation. Quantifying inflammatory reactions, such as anterior chamber cell counts, using AS-OCT has shown clear advantages, particularly in uveitis.^11^^,^^19^ Further progress will require more automated assessment methods.

The LPI-induced changes in the vessel density and perfusion area were quantified by AS-OCTA. This is the first time in the literature that localized vascular changes after the LPI have been reported. However, given that the vessel density and perfusion area showed similar trends in both groups, that is, rapidly increasing after LPI and subsiding at 1 day and 1 week (see Fig. 3), we suspected that these might represent temporary changes secondary to LPI. Similar to the laser treatment in the fundus,^20^ the laser beam might induce a localized acute congestion, followed by a recovery of the vascular structure. However, the exact effect of these vascular changes remains uncertain and requires longitudinal observation. In addition, the larger area of LPI was significantly related to a lower perfusion area, as such, we recommended a smaller LPI site in the clinical procedure.

In the current study, AOD750 was selected as the primary angle parameter as it is more strongly correlated with gonioscope angle assessment than other AS-OCT parameters.^12^^,^^21^ As shown in Figure 3, the AS-OCTA–guided group had a significantly larger AOD750 than the slit lamp-guided group. It was likely due to more superior LPI sites in the AS-OCTA–guided group, which led to a higher extent of angle opening. The reason why the superior sites are more likely to respond to LPI has not been clear until now.^12^^,^^22^

There are some limitations in this study. First, the sample size was relatively small, and future research should include more patients with different grades of PAC disease. Second, we did not perform AS-OCTA–guided LPI and slit lamp-guided LPI under a double-blind protocol. Third, and most importantly, there was no follow-up mode in the anterior segment scanning; therefore, the vascular assessment relied on a manual registration process (by author J.L.), which might have some bias in interpreting the vascular changes at the LPI sites and their parameters. Besides, the LPI site was selected clinically in the slit lamp group and was not standardized to a specified region, which might introduce bias and potential differences in the widening of the anterior chamber angle.^1^

In summary, the incidence of anterior chamber bleeding could be lowered by knowing the iris vessel layout measured using AS-OCTA before the LPI procedure. AS-OCT/OCTA may also be used for immediate assessment and follow-up of LPI treatment efficacy and anterior chamber responses.
